# Standardisation of defined approaches for skin sensitisation testing to support regulatory use and international adoption: position of the International Cooperation on Alternative Test Methods

**DOI:** 10.1007/s00204-017-2097-4

**Published:** 2017-11-10

**Authors:** S. Casati, K. Aschberger, J. Barroso, W. Casey, I. Delgado, T. S. Kim, N. Kleinstreuer, H. Kojima, J. K. Lee, A. Lowit, H. K. Park, M. J. Régimbald-Krnel, J. Strickland, M. Whelan, Y. Yang, Valérie Zuang

**Affiliations:** 1 European Commission, Joint Research Centre (JRC), 21027 Ispra, Italy; 2 National Toxicology Program Interagency Center for the Evaluation of Alternative Toxicological Methods, National Institute of Environmental Health Sciences, Research Triangle Park, Morrisville, NC 27709 USA; 3 BraCVAM, National Institute of Quality Control in Health, Oswaldo Cruz Foundation, Rio de Janeiro, Brazil; 4 Korean Center for the Validation of Alternative Methods, National Institute of Food and Drug Safety Evaluation, Cheongju, Chungcheongbuk-do Republic of Korea; 5 Japanese Center for the Validation of Alternative Methods, National Institute of Health Sciences, Tokyo, 158-8501 Japan; 6 Office of Pesticide Programs, U.S. Environmental Protection Agency, Washington DC, 20460 USA; 7 Environmental Health Science and Research Bureau, Healthy Environments and Consumer Safety Branch, Health Canada, Ottawa, ON K1A 0K9 Canada; 8 Integrated Laboratory Systems inc., Research Triangle Park, Morrisville, NC 27709 USA; 9 Institute of Toxicology, Guangdong Provincial Center for Disease Control and Prevention, Guangzhou, 510300 China

**Keywords:** Skin sensitisation, Defined approaches, Alternative methods, International standards, Adverse outcome pathway

## Abstract

Skin sensitisation is the regulatory endpoint that has been at the centre of concerted efforts to replace animal testing in recent years, as demonstrated by the Organisation for Economic Co-operation and Development (OECD) adoption of five non-animal methods addressing mechanisms under the first three key events of the skin sensitisation adverse outcome pathway. Nevertheless, the currently adopted methods, when used in isolation, are not sufficient to fulfil regulatory requirements on the skin sensitisation potential and potency of chemicals comparable to that provided by the regulatory animal tests. For this reason, a number of defined approaches integrating data from these methods with other relevant information have been proposed and documented by the OECD. With the aim to further enhance regulatory consideration and adoption of defined approaches, the European Union Reference Laboratory for Alternatives to Animal testing in collaboration with the International Cooperation on Alternative Test Methods hosted, on 4–5 October 2016, a workshop on the international regulatory applicability and acceptance of alternative non-animal approaches, i.e., defined approaches, to skin sensitisation assessment of chemicals used in a variety of sectors. The workshop convened representatives from more than 20 regulatory authorities from the European Union, United States, Canada, Japan, South Korea, Brazil and China. There was a general consensus among the workshop participants that to maximise global regulatory acceptance of data generated with defined approaches, international harmonisation and standardisation are needed. Potential assessment criteria were defined for a systematic evaluation of existing defined approaches that would facilitate their translation into international standards, e.g., into a performance-based Test Guideline. Informed by the discussions at the workshop, the ICATM members propose practical ways to further promote the regulatory use and facilitate adoption of defined approaches for skin sensitisation assessments.

## Background

Skin sensitisation is a regulatory endpoint required for many chemical sectors (e.g., industrial chemicals, pesticides, and cosmetics) and has been at the centre of concerted efforts to replace animal testing in recent years. The European Union Reference Laboratory for Alternatives to Animal Testing (EURL ECVAM) of the European Commission’s Joint Research Centre, with the implementation of its strategy for the skin sensitisation area (EURL ECVAM 2013), played a pivotal role in assuring the translation of well-established non-animal methods into internationally adopted Test Guidelines (TG). As a result, between 2015 and 2017, in chemico and in vitro test methods addressing mechanisms under the first three key events of the skin sensitisation adverse outcome pathway (AOP) (OECD 2012) have been adopted by the Organisation for Economic Co-operation and Development (OECD). In addition to the adopted Direct Peptide Reactivity Assay (DPRA, OECD TG 442C) (OECD 2015a), the ARE-Nrf2 Luciferase Test Method (KeratinoSens^™^, OECDTG 442D) (OECD 2015b), the human Cell Line Activation Test (h-CLAT), the U-SENS^™^ and the IL-8 Luc assay, the latter three all described in OECD TG 442E (OECD 2017a); other in vitro methods, such as the LuSens, the SENS-IS^™^ and the GARD assays, have been included in the OECD TG work programme.

The currently adopted test methods, when used in isolation, are not able to fulfil all regulatory requirements on the skin sensitisation potential and potency of chemicals comparable to that provided by the regulatory animal tests, i.e., the Local Lymph Node Assay (LLNA) (OECD TG 429) (OECD 2010a) or its non-radioactive variants, LLNA: DA (OECD TG 442A) (OECD 2010b) and LLNA: BrdU-ELISA (OECD TG 442B) (OECD 2010c), and the Guinea Pig Maximisation Test (GPMT) and Buehler Test (both described in OECD TG 406) (OECD 1992). For this reason, data generated with the DPRA, the ARE-Nrf2 Luciferase Test Method (KeratinoSens^™^) and the three methods addressing dendritic cell activation (h-CLAT, U-SENS^™^ and IL-8 Luc Assay) should be considered in the context of integrated approaches to testing and assessment (IATA), in combination with other relevant complementary information if available, e.g., physical–chemical properties, information on other key events of the skin sensitisation AOP as well as non-testing methods, including read-across from chemical analogues.

Over the past few years, a number of defined approaches (DAs) integrating information from multiple non-animal methods (e.g., in silico, in chemico, in vitro) and other relevant information (e.g., physico-chemical properties) have been developed for the purpose of skin sensitisation hazard assessment and/or potency categorisation. In 2013, as a first step in promoting their regulatory implementation at an international level, EURL ECVAM, on behalf of the European Commission, made a project proposal to the OECD for the development of guidance to ensure harmonised reporting of DAs that would ultimately facilitate their evaluation and application in IATA for regulatory purposes. Charged with the definition of such guidance was an expert group run, at the time, under the OECD Task Force on Hazard Assessment, which is now called the Working Party on Hazard Assessment. This work resulted in the publication in 2016 of two OECD guidance documents (GD) (GD 255 and GD 256, OECD 2016a, b, c) on the harmonised reporting of DAs. Although the guidance on harmonised reporting provides a standardised format for describing DA elements and reporting performance that is practical for regulatory review, it does not guarantee actual deployment of DAs or acceptance of DA predictions by regulatory bodies in the various regions.

## IATA and defined approaches

An important concept put forward by the OECD is the distinction between IATA and DAs. IATA are defined as pragmatic, science-based approaches for chemical hazard or risk assessment that rely on an integrated analysis of existing information coupled with the generation of new information using testing strategies. IATA follow an iterative approach to answer a defined question in a specific regulatory context, taking into account the acceptable level of uncertainty associated with the decision context (OECD 2017b). The overall assessment process within IATA is based on weight-of-evidence (WoE), which necessarily implies an expert judgment in the weighing of the different pieces of information.

Non-animal approaches developed in the area of skin sensitisation that is based on a fixed set of information sources and fixed data interpretation procedure are designated as “defined approaches to testing and assessment” (OECD 2016a). The DA designation emphasises that predictions generated by these approaches are rule-based and are not influenced by expert judgment. The fixed nature of DAs should facilitate their consideration under the OECD mutual acceptance of data (MAD), whereas IATA are designed to be flexible and adaptable to particular regional requirements or regulatory statutes.

**Fig. 1:**
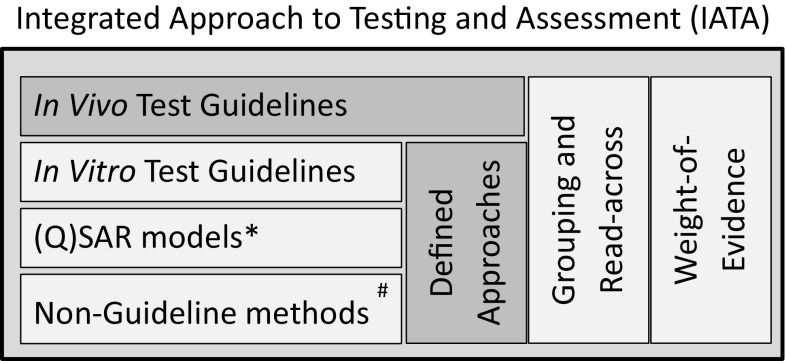
Generic IATA elements and role of defined approaches within IATA. *Quantitative structure–activity (QSAR) models are usually characterised according to the five OECD principles for QSAR model validation (OECD 2007). ^#^Non-guideline in vitro test methods should be described according to OECD Guidance Document No. 211

Figure 1 provides a generic representation of IATA and its elements. It illustrates how predictions obtained with DAs that integrate non-animal data can be used to support the WoE assessment within IATA when used together with other relevant information, including in vivo data, if these already exist. DAs may also be considered equivalent alternatives to in vivo data if providing the same level of information within the decision context of the IATA.

Some of the DAs for skin sensitisation seem to be as good as, if not better than, the LLNA in predicting skin sensitisation responses in humans (EURL ECVAM 2017); as such, they may be used as valid components of IATA (e.g., together with all existing reliable and relevant information) for skin sensitisation assessment as alternatives to LLNA data or in conjunction with these data if they already exist. Moreover, it is envisaged that some of the individual test methods currently under development or formal evaluation (i.e., validation and/or peer review and included in the OECD TG programme) may have similar or better performance than the LLNA.

## ICATM workshop on the international regulatory applicability and acceptance of alternative non-animal approaches to skin sensitisation

With the aim to further enhance regulatory consideration and adoption of individual test methods and DAs, EURL ECVAM in collaboration with the International Cooperation on Alternative Test Methods (ICATM),^1^ hosted a 2-day workshop (4th–5th October 2016) on the international regulatory applicability and acceptance of alternative non-animal approaches to skin sensitisation assessment of chemicals used in a variety of sectors.

The workshop convened representatives from more than 20 regulatory authorities from the European Union (EU), United States (US), Canada, Japan South Korea, Brazil, and China, to facilitate a common understanding of the available non-animal methods (i.e., in vitro, in chemico, in silico and read-across) and their role within DAs. Working together to identify potential obstacles, the international and cross-sector group defined a series of steps that should be taken to support the regulatory application of DAs.

The participants initially focused on the current regulatory requirements for skin sensitisation in different regions by chemical sector (i.e., pesticides, cosmetics, pharmaceuticals, industrial chemicals, etc.). Although it was recognised that there are differences in requirements by the various jurisdictions and sectors, opportunities to satisfy regulatory requirements with the use of individual non-animal methods and DAs were also highlighted (Daniel et al. 2017).

There was general consensus among the workshop participants that to maximise regulatory consideration and acceptance of data generated with DAs, international harmonisation and standardisation will be necessary. This should ideally be achieved through the development of an evaluation framework that allows an independent assessment of the DAs currently reported in Annex I to OECD GD 256 (OECD 2016b) and any other upcoming promising DAs.

Possible criteria for the evaluation framework were proposed and discussed at the workshop. According to the workshop participants, the criteria should be based on key elements, such as biological plausibility, inclusion of existing validated in vitro methods covering AOP key events, accessible data interpretation procedures, and performance using reference chemicals. Another important aspect raised was the fact that when judging the predictive capacity of a DA against reference animal data, the DA should not be expected to show better predictivity than the performance of the animal test in predicting itself (reproducibility of the reference animal data). These criteria are further detailed below.

The participants also agreed that the OECD would, at the current time, represent the most suitable forum for further evaluating DAs and achieving international acceptance through their translation into international standards, e.g., into a performance-based Test Guideline.

## Initial criteria proposed for the assessment of defined approaches

At the ICATM workshop, participants discussed potential criteria that could be used for the assessment of DAs for skin sensitisation. Although thorough international discussion and agreement of such criteria will occur during the OECD process for drafting a performance-based Test Guideline (PBTG), ICATM partners agreed that the following proposed criteria are extremely relevant and should be considered in the development of the evaluation framework:

- The reproducibility of a DA should provide a level of confidence no less than that provided by the reproducibility of the reference animal test.
- The relevance/predictive capacity of a DA should be compared to the predictive capacity of the animal test to predict human responses if high quality reference human data are available. When human data are not available and thus judging the predictive capacity of a DA directly against reference animal data is necessary, the DA should not be expected to show better predictivity than the animal test is able to predict itself.
- The DA should provide an equivalent level of information as the reference animal test method, depending on the decision context of the sector/regulatory framework. For example, the DA should at minimum provide hazard information and should ideally provide sufficient information for classification and labelling.
- The DA should be mechanistically and biologically relevant, preferably with respect to an existing AOP framework. The DA should cover at least one molecular initiating event or key event of the AOP.
- The DA should be transparently described using the template provided in OECD GD 255 (OECD 2016a, b, c) (e.g., the chemical space/applicability domain for which the DA works and its known limitations must be clearly described, including applicability to multi-constituent substances, mixtures, substances of unknown and variable composition, etc.) (OECD 2016c). OECD GD 255 also recommends that if non-OECD TG methods are used, they should be reported according to OECD GD 211 and in silico models should be characterised according to the five OECD principles for QSAR model validation (OECD 2014) and reported using the QSAR Model Reporting Format, accessible at: https://eurl-ecvam.jrc.ec.europa.eu/laboratories-research/predictive_toxicology/qsar_tools/qrf.
- Independent evaluation and implementation by third parties must be possible (i.e., all of the DA components must be readily accessible and all the relevant protocols must be available).
- Ideally, the DA should include one or more OECD TG methods to facilitate acceptance.
- Conflicting results between reference in vivo data and DA information sources should be properly discussed and, if possible, explained.
- Uncertainty (both at the level of the DA and of the reference data against which the DA is assessed) should be described to the fullest extent possible.
- The DA and its individual information sources should undergo a quality assured, independent scientific review to raise confidence in the approach.
- Criteria for selecting reference chemicals should be defined for the particular regulatory area, to cover the relevant applicability domain, rather than developing one definitive list.
- DA predictions should be considered in the context of IATA together with all available and relevant information, when available.

## ICATM position on the standardisation of defined approaches

Informed by the discussions at the workshop, the ICATM members met on 6 October 2016 to discuss practical ways to further promote the regulatory use of DAs in the area of skin sensitisation. The main action discussed and agreed upon was the submission to the OECD of a project proposal for the development of a PBTG for DAs for skin sensitisation testing and assessment. Such a proposal (co-led by the EU, US and Canada) was submitted to the OECD in November 2016, and following revision based on feedback from Member Countries, was approved on 27 April 2017 for inclusion in the OECD workplan.

As suggested by the ICATM workshop participants, the primary milestone within the project would be the development of an evaluation framework that would allow a consistent assessment of DAs for skin sensitisation. Acceptable performance of DAs will be defined on the basis of a comprehensive characterisation of the reproducibility of the LLNA and its relevance for predicting human skin sensitisation potential and potency. A number of publications that have analysed the variability in the animal data, including ICCVAM 2011, Hoffman 2015, Roberts et al. 2016, and Dumont et al. 2016, will be included in a meta-analysis of the literature. The human data will be collected from different sources (e.g., Basketter et al. 2014; Bell et al. 2017, etc.), and the performance of the LLNA against human data will be compared on the basis of as many chemicals with reliable data as possible.

The resulting PBTG will comprise DAs and possibly individual non-animal methods that have been shown to meet the criteria defined in the evaluation framework and that provide the same level of information or are more informative than the LLNA for human hazard identification (i.e., sensitiser versus non-sensitiser) and/or classification and labelling of chemicals [e.g., according to the United Nations Globally Harmonised System of Classification and Labelling of Chemicals (GHS) Category 1, 1A and 1B] (UN 2011).

The ICATM partners agreed that the development of an OECD PBTG for DAs would facilitate their inclusion in the Mutual Acceptance of Data (MAD) agreement (OECD 1981), which requires test data generated in any OECD member country to be accepted in other member countries. This would overcome some of the obstacles currently associated with the acceptance of non-animal data by:

- Increasing the confidence of regulators in such approaches.
- Facilitating implementation and use by industries and contract research organisations.
- Reducing duplicate testing and therefore costs.

The definitions of DA and IATA, and how these could be better described and presented to increase understanding and drive progress, were also addressed during the ICATM discussion. The decision was made to create informational materials to bring all those involved in this area to the same level of understanding.

## Additional identified mid- and long-term actions to further promote the implementation, use and acceptance of non-animal approaches for skin sensitisation

A number of diverse stakeholders were identified as critical to advancing progress in the area of DAs for skin sensitisation testing and assessment. Many researchers from industry, academic institutions, and non-governmental organisations have experience with the in vitro and in silico test methods that form the building blocks of DAs. Companies use these non-animal test methods early in their product development pipelines to identify promising candidates, but often these data are never submitted to regulatory authorities in those regions where such data are not mandatory for regulatory purposes. ICCVAM agencies such as the US Environmental Protection Agency, and other ICATM partners, have initiated several voluntary data sharing pilots to encourage industry stakeholders to make such internal resources available so that the applicability domain and performance of non-animal methods can be assessed for broad areas of toxicology (e.g., https://ntp.niehs.nih.gov/pubhealth/evalatm/test-method-evaluations/acute-systemic-tox/iv-data-req/index.html). Testing laboratories that are engaged in commercialising the in vitro methods with associated OECD TGs also have valuable implementation experience that could be shared with the broader scientific community. Knowledge sharing among research stakeholders will create efficient use of limited resources and accelerate progress towards full characterisation of DAs.

On the regulatory side, scientists are often unfamiliar with the existence, let alone the performance and validation status, of new approaches. Training and educational materials need to be developed to spread awareness of DAs and where they may be appropriate to fulfil regulatory requirements. Increased familiarity with new tools and data sources will allow regulators to gain confidence in evaluating the output of DAs included in regulatory submission packages. The necessity for increased communication also applies in the reverse: agencies that are open to accepting non-animal test data should publish guidance for registrants and offer training to industry as well as their own scientists.

To enable wider regulatory uptake of DAs, their applicability to specific chemical/product classes should be further characterised. The majority of existing data generation efforts and analyses comparing DAs to animal test data for skin sensitisation have been focused on personal care product and cosmetics ingredients, largely motivated by the EU ban on animal testing for marketed chemicals in that sector. Work has been done to show that many of these chemicals have multiple uses and are relevant to other sectors, e.g., industrial chemical manufacturing, but to ensure coverage of the relevant chemical space for many regulatory bodies, data generated on a broad range of chemistries are needed, ideally including formulations. Organisations such as the U.S. National Toxicology Program (NTP) are funding efforts to test chemicals and products (with existing in vivo data) nominated by regulatory and research agencies in three of the in vitro assays with existing OECD TGs. These data will be used to further explore the performance and applicability for a broader chemical/product space of the DAs published in Kleinstreuer et al. (2017).

ICATM partners agreed that the ultimate common goal among countries and agencies is the international harmonisation of regulatory requirements that would allow the use of DAs instead of the current regulatory animal tests.

To achieve this goal, work is ongoing with the OECD to develop an evaluation framework for DAs for skin sensitisation. DA fulfilling the evaluation criteria would qualify for inclusion in a PBTG on DA and would fall under the OECD’s MAD agreement. This will motivate OECD member countries to accept assessments generated according to the PBTG. Multi-stakeholder dialogues and international collaborations to share knowledge and experience will ultimately increase confidence and facilitate the widespread implementation and regulatory use of non-animal approaches.

## Conclusions

Considerable progress has been made in the area of non-animal methods for skin sensitisation assessment. Integration of data generated by these methods in DAs has the potential to replace to a large extent the use of the current animal models. The ICATM members agreed that an evaluation framework should be developed that allows an independent assessment of the DAs currently reported in Annex I to OECD GD 256 (OECD 2016b) and any other upcoming promising DAs. The DAs that would qualify against this evaluation framework should be translated into international standards allowing the predictions obtained with these DAs to fall under the OECD MAD agreement and thus substantially obviate the need to generate new animal data to fulfil regulatory obligations for skin sensitisation within OECD member countries. Work has already started at the OECD level with the leadership of EU, US and Health Canada and support from the other ICATM partners, for the development of such an evaluation framework.

## Footnotes

1 ICATM is the "International Cooperation on Alternative Test Methods" consisting of the European Union Reference Laboratory for Alternatives to Animal Testing (EURL ECVAM), the US Interagency Coordinating Committee on the Validation of Alternative Methods (ICCVAM), the Japanese Centre for the Validation of Alternative Methods (JaCVAM), the Korean Center for the Validation of Alternative Methods (KoCVAM), and Health Canada. Although not yet formally partners, the Brazilian Center for the Validation of Alternative Methods (BraCVAM) and China also actively participate. ICATM partners are working together to promote enhanced international cooperation and coordination on the scientific development, validation and regulatory use of alternative approaches. Additional information on ICATM can be found at: https://eurl-ecvam.jrc.ec.europa.eu/about-ecvam/networks-and-collaborations/collaboration-with-icatm.
